# Attachment of Li[Ni_0.2_Li_0.2_Mn_0.6_]O_2_ Nanoparticles to the Graphene Surface Using Electrostatic Interaction Without Deterioration of Phase Integrity

**DOI:** 10.1186/s11671-016-1483-9

**Published:** 2016-05-27

**Authors:** Min Ho Pyun, Yong Joon Park

**Affiliations:** Department of Advanced Materials Engineering, Kyonggi University, San 94-6, Iui-dong, Yeongtong-gu, Suwon-si, Gyeonggi 443-760 South Korea

**Keywords:** Nanoparticle, Graphene, Rate capability, Cathode, Li battery, Composition

## Abstract

In this article, we report a facile approach to enhance the electrochemical performance of Li-rich oxides with vulnerable phase stability. The Li-rich oxide nanoparticles were attached to the surface of graphene; the graphene surface acted as a matrix with high electronic conductivity that compensated for the low conductivity and enhanced the rate capability of the oxides. Our novel approach constitutes a direct assembly of two materials via electrostatic interaction, without a high-temperature heat treatment. The inevitable deterioration in phase integrity of previous composites between carbon and Li-rich oxides resulted from the reaction of oxygen in the structure with carbon during the heat-treatment process. However, our new method successfully attached Li-rich nanoparticles to the surface of graphene, without a phase change of the oxides. The resulting graphene/Li-rich oxide composites exhibited superior capacity and rate capability compared to their pristine Li-rich counterparts.

## Background

Recently, Li-ion batteries have been used as the main energy storage system for electrical devices and electrical vehicles [1–5]. However, the energy densities of commercial Li-ion batteries using LiCoO_2_ as a cathode cannot satisfy the demand of many of these applications [6–10]; new high-capacity cathode materials are therefore required. The layered Li-rich materials in the Li-Mn-Ni oxide system, which have higher energy densities (~250 mAh g^−1^) than other cathode materials such as LiCoO_2_ [11–18], have attracted significant attention as promising new cathode materials. However, major drawbacks such as poor rate capability owing to insufficient electronic and ionic conductivities [19–21] have prevented their use in commercial applications. Many attempts have been made to enhance the rate capability of the cathode-like Li-rich layered oxide. For example, compositing the oxide with carbon-based materials that have high electronic conductivity has been used as a means of compensating for the low conductivity of the cathode materials [22–25]. In fact, graphene comprising two-dimensional carbon sheets has been used as a novel matrix in cathode/carbon composites that have high rate capability and stable cyclic performance; the use of graphene stems from its excellent electronic conductivity and mechanical flexibility as well as high specific surface area [26–29]. However, compositing with carbon materials (such as graphene) requires high-temperature (greater than ~400 °C) heat-treatment processes that produce strong bonds between the cathode and carbon. Unfortunately, Li-rich layered oxides are susceptible to phase changes and deterioration of the structural integrity during processing; this deterioration results from the reaction of carbon with oxygen (at over 400 °C) and the consequent loss of oxygen from the structure of the oxides [28, 29]. Since most of the cathode materials, such as LiCoO_2_, LiFePO_4_, and LiMn_2_O_4_, are relatively stable during the heating process [22–27], this susceptibility to deterioration is attributed to the structural instability of the oxides.

**Fig. 1:**
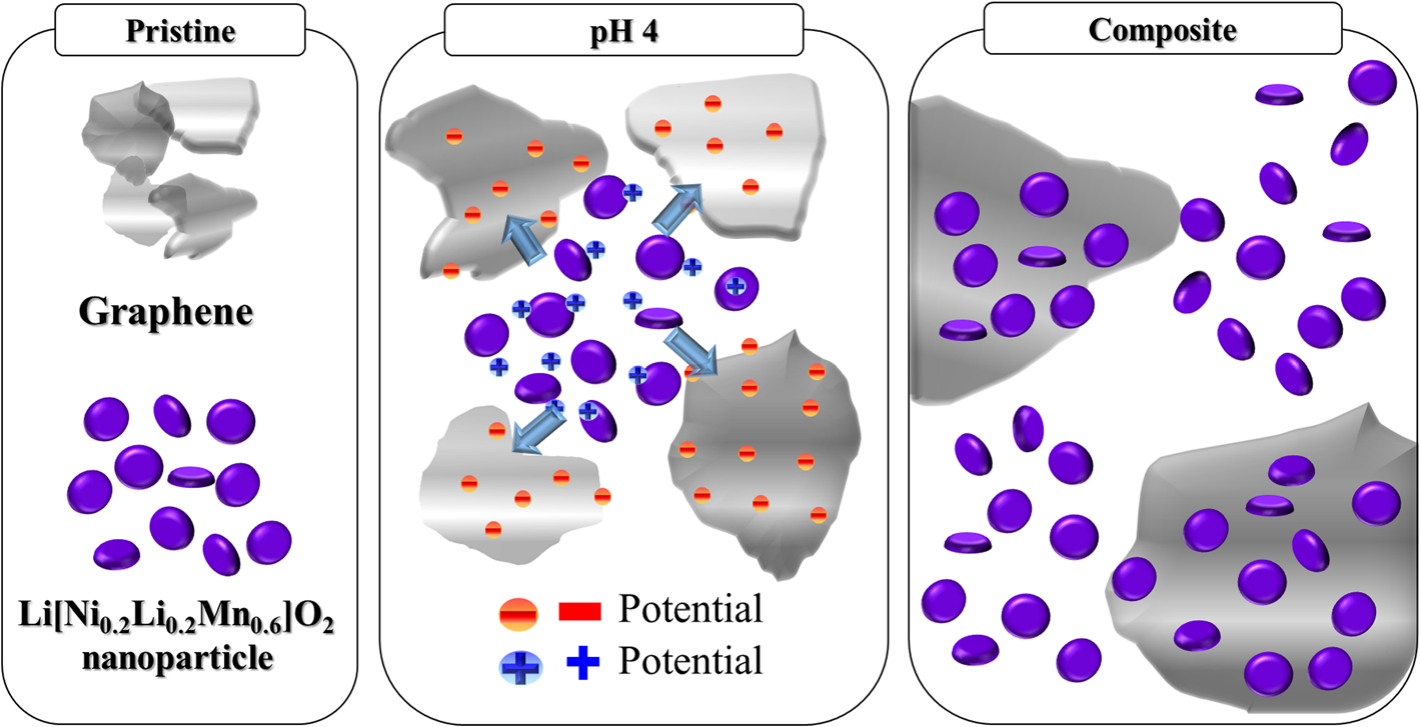
Schematic showing the fabrication process of graphene/Li[Ni_0.2_Li_0.2_Mn_0.6_]O_2_ nanoparticle composites via electrostatic interaction

To overcome this drawback, we propose a new method for the heat-treatment-free fabrication of composites between graphene and unstable Li-rich layered oxides. Our novel strategy for the composition relies on the electrostatic interaction of the two species. As illustrated in Fig. 1, the graphene particles and Li-rich layered oxides can be negatively and positively charged, respectively, by controlling the pH of the solution. These inversely charged particles induce a self-assembly between the graphene and the oxides via electrostatic interactions. This method is especially attractive because the graphene/cathode composite can be prepared through a simple drying process, without a high-temperature heat treatment. As such, the loss of oxygen is prevented and the phase and structural integrity of the vulnerable Li-rich layered oxides are maintained. In this work, Li[Ni_0.2_Li_0.2_Mn_0.6_]O_2_ nanoparticles (a typical Li-rich layered oxide) attached to the graphene surface were prepared via electrostatic interaction and tested as a cathode material for enhanced Li batteries.

## Methods

Li[Ni_0.2_Li_0.2_Mn_0.6_]O_2_ nanoparticles were prepared via the general combustion method using a dispersing agent to control the particle size of the cathode powder [21]. The prepared Li[Ni_0.2_Li_0.2_Mn_0.6_]O_2_ nanoparticles were composited with graphene via electrostatic interactions. The commercial graphene (AMG Graphite/Graphit Kropfmühl) was immersed into the 1-N acidic solution (nitric acid/sulfuric acid = 3:1) for 2 h for surface modification and washed several times using distilled water. Then the Li[Ni_0.2_Li_0.2_Mn_0.6_]O_2_ and graphene particles were dispersed in an ethanol-based solution by ultra-sonication; the weight % of the graphene to the Li[Ni_0.2_Li_0.2_Mn_0.6_]O_2_ was controlled to amounts of 0.5, 2.0, and 5.0. The pH of the solution was adjusted to 4 using buffer solution (pH 4) in order to control the surface potential of the graphene and the Li[Ni_0.2_Li_0.2_Mn_0.6_]O_2_ particles. After a 10-min stirring for homogeneous mixing, the solution was vacuum filtered and the resulting samples were dried at 200 °C for 2 h under an air atmosphere. X-ray diffraction (XRD) measurements were then performed on the samples with a Rigaku X-ray diffractometer using monochromatized Cu-K_α_ radiation (*λ* = 1.5406 Å). In addition, the surface of the Li[Ni_0.2_Li_0.2_Mn_0.6_]O_2_ nanoparticle/graphene composite was examined using a transmission electron microscope (TEM, JEOL-4010) and X-ray photoelectron spectroscopy (XPS, PHI 5000 VersaProbe, Ulvac-PHI). The C content of the composites was determined via thermogravimetric analysis (TGA, Q500 V20.13 Build 39) by heating from 200 to 750 °C under an air atmosphere.

For electrochemical testing, a cathode slurry was prepared by mixing Li[Ni_0.2_Li_0.2_Mn_0.6_]O_2_ nanoparticle/graphene composite (or pristine Li[Ni_0.2_Li_0.2_Mn_0.6_]O_2_ nanoparticles) and carbon black (Super P) with polyvinylidene fluoride (PVDF) in a weight ratio of 80 (cathode):10 (super P):10(PVDF). After 24 h of ball-mill processing, the viscous slurry was coated onto an Al foil using a doctor blade and subsequently dried at 90 °C in an oven. A coin-type cell (2032) consisting of a cathode, Li-metal anode, separator (25 μm, SK Innovation), and an electrolyte (1 M LiPF_6_ in EC/DMC (1:1 vol%)) was used. The cells were subjected to galvanostatic cycling in the voltage range of 4.8–2.0 V and at various charge-discharge rates, using a WonATech voltammetry system. In addition, impedance measurements were performed by applying an AC voltage at an amplitude of 5 mV and a frequency range of 0.1 Hz to 100 KHz, using an electrochemical workstation (AMETEK, VersaSTAT 3).

## Results and Discussion

**Fig. 2:**
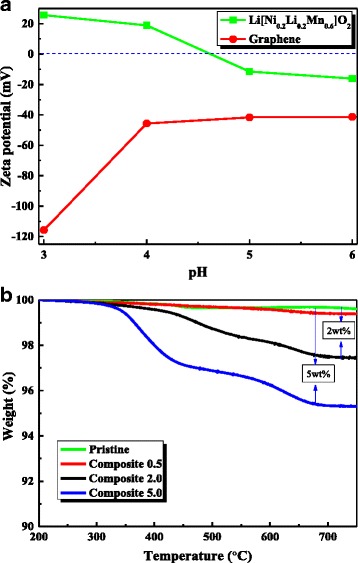
**a** The zeta potential of the graphene and Li[Ni_0.2_Li_0.2_Mn_0.6_]O_2_ nanoparticle surface as a function of the pH value; **b** results from the TGA of the pristine and composite samples

An appropriate pH value must be selected in order to directly assemble composites of graphene and Li[Ni_0.2_Li_0.2_Mn_0.6_]O_2_ nanoparticles via electrostatic interaction. Therefore, the appropriate pH value of the surface charges of graphene and the Li[Ni_0.2_Li_0.2_Mn_0.6_]O_2_ nanoparticles was determined via zeta potential measurements. As Fig. 2a shows, the surface of graphene was negatively charged at pH values of 3–6. In contrast, the surface charge of the Li[Ni_0.2_Li_0.2_Mn_0.6_]O_2_ nanoparticle switched from positive (over 20 mV) to negative (−20 mV) with increasing pH value. The graphene and Li[Ni_0.2_Li_0.2_Mn_0.6_]O_2_ nanoparticles should be oppositely charged in order to trigger the mutual assembly via electrostatic interaction, and hence the pH value should be lower than 4. Figure 2a shows that the electrostatic interaction between the particles increased with decreasing pH value. However, the low pH resulted in reduced integrity of the nanoparticles owing to the vulnerable surface of the Li-rich oxide in the acidic environment. As such, we determined that a pH value of 4 was appropriate, because this was the highest value that allowed the formation of composites between the oppositely charged graphene and Li[Ni_0.2_Li_0.2_Mn_0.6_]O_2_ nanoparticles.

To determine the actual graphene content, the results of the TGA of the pristine and composite samples were measured and compared as shown in Fig. 2b. If the composite sample is prepared through a high-temperature heat treatment, then some of the graphene will evaporate during the process. However, our composite samples were dried at 200 °C without heat treatment, and hence the original amount of graphene was maintained during the fabrication process. As Fig. 2b shows, composite 0.5, composite 2.0, and composite 5.0 exhibit weight loss of ~0.5, 2.0, and 5.0 wt.%, respectively, when the samples are heated to 750 °C. This weight loss stems from the evaporation of carbon and is therefore an indicator of the carbon content of the composites.

**Fig. 3:**
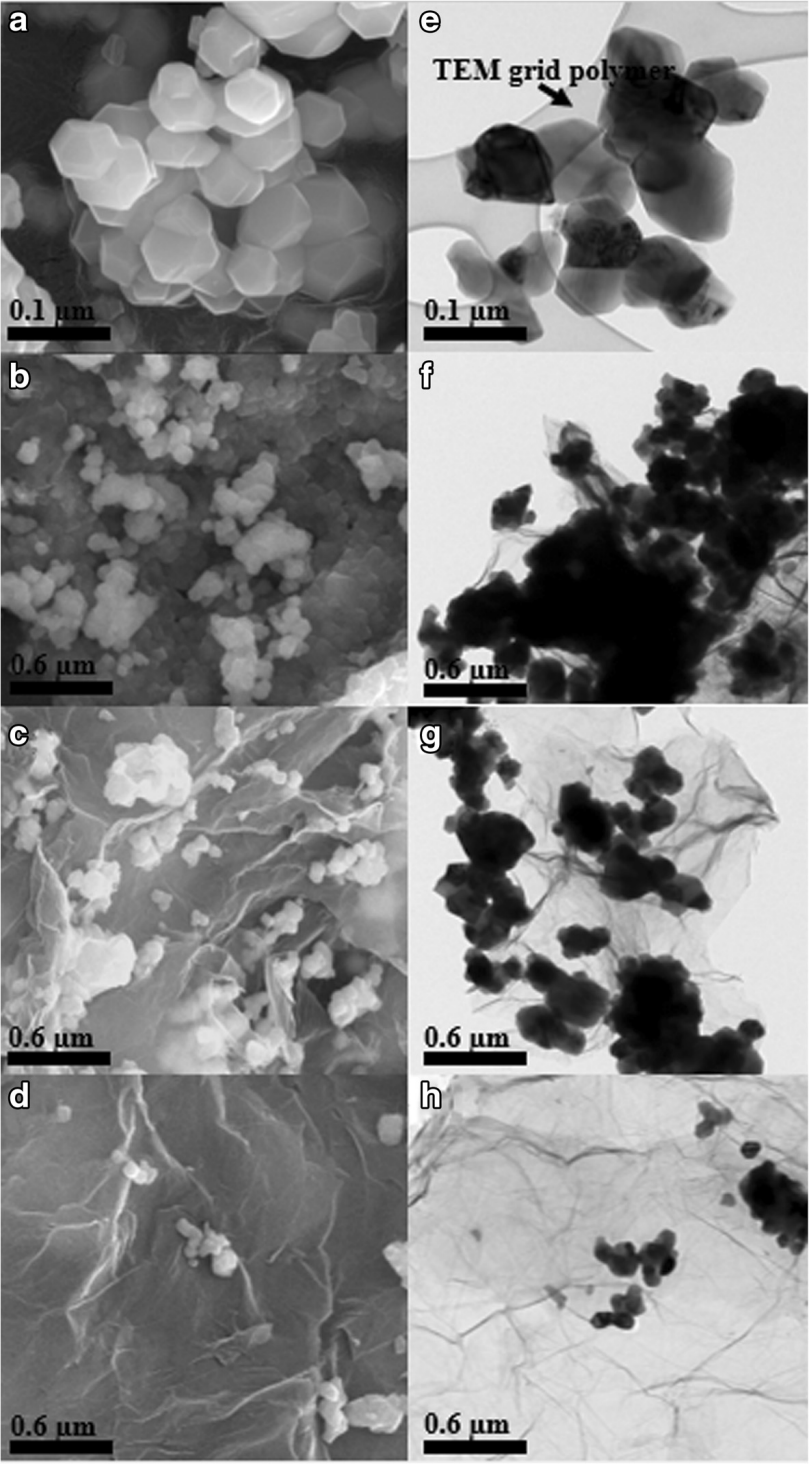
SEM images of the **a** Li[Ni_0.2_Li_0.2_Mn_0.6_]O_2_ nanoparticle, **b** composite 0.5, **c** composite 2.0, and **d** composite 5.0; TEM images of the **e** Li[Ni_0.2_Li_0.2_Mn_0.6_]O_2_ nanoparticle, **f** composite 0.5, **g** composite 2.0, and **h** composite 5.0

Figure 3 shows SEM and TEM images of the pristine Li[Ni_0.2_Li_0.2_Mn_0.6_]O_2_ nanoparticles and composites of the graphene and Li[Ni_0.2_Li_0.2_Mn_0.6_]O_2_ nanoparticles. Hereafter, we refer to the Li[Ni_0.2_Li_0.2_Mn_0.6_]O_2_ nanoparticles attached to 0.5 wt.% graphene, 2.0 wt.% graphene, and 5.0 wt.% graphene (wt.% means weight percent of the Li[Ni_0.2_Li_0.2_Mn_0.6_]O_2_ nanoparticles) as composite 0.5, composite 2.0, and composite 5.0, respectively. The Li[Ni_0.2_Li_0.2_Mn_0.6_]O_2_ powder consists of 200~500-nm-sized nanoparticles (Fig. 3a, e). Figure 3b, f shows that composite 0.5 consists of Li[Ni_0.2_Li_0.2_Mn_0.6_]O_2_ particles that are successfully composited with graphene; i.e., the particles cover most of the graphene surface. However, many of the particles were aggregated and did not have direct contact with graphene owing to its low (only 0.5 wt.%) surface area. In contrast, the graphene surface of composite 2.0 was appropriately covered with Li[Ni_0.2_Li_0.2_Mn_0.6_]O_2_ nanoparticles, as shown in Fig. 3c, g. The nanoparticles seemed to be strongly attached to graphene, which has a high electronic conductivity. Figure 3d, h shows the image of composite 5.0. As the figure shows, the Li[Ni_0.2_Li_0.2_Mn_0.6_]O_2_ nanoparticles are well-attached to, but only sparsely populate graphene; i.e., the surface area of the graphene far exceeded that of the nanoparticles

**Fig. 4:**
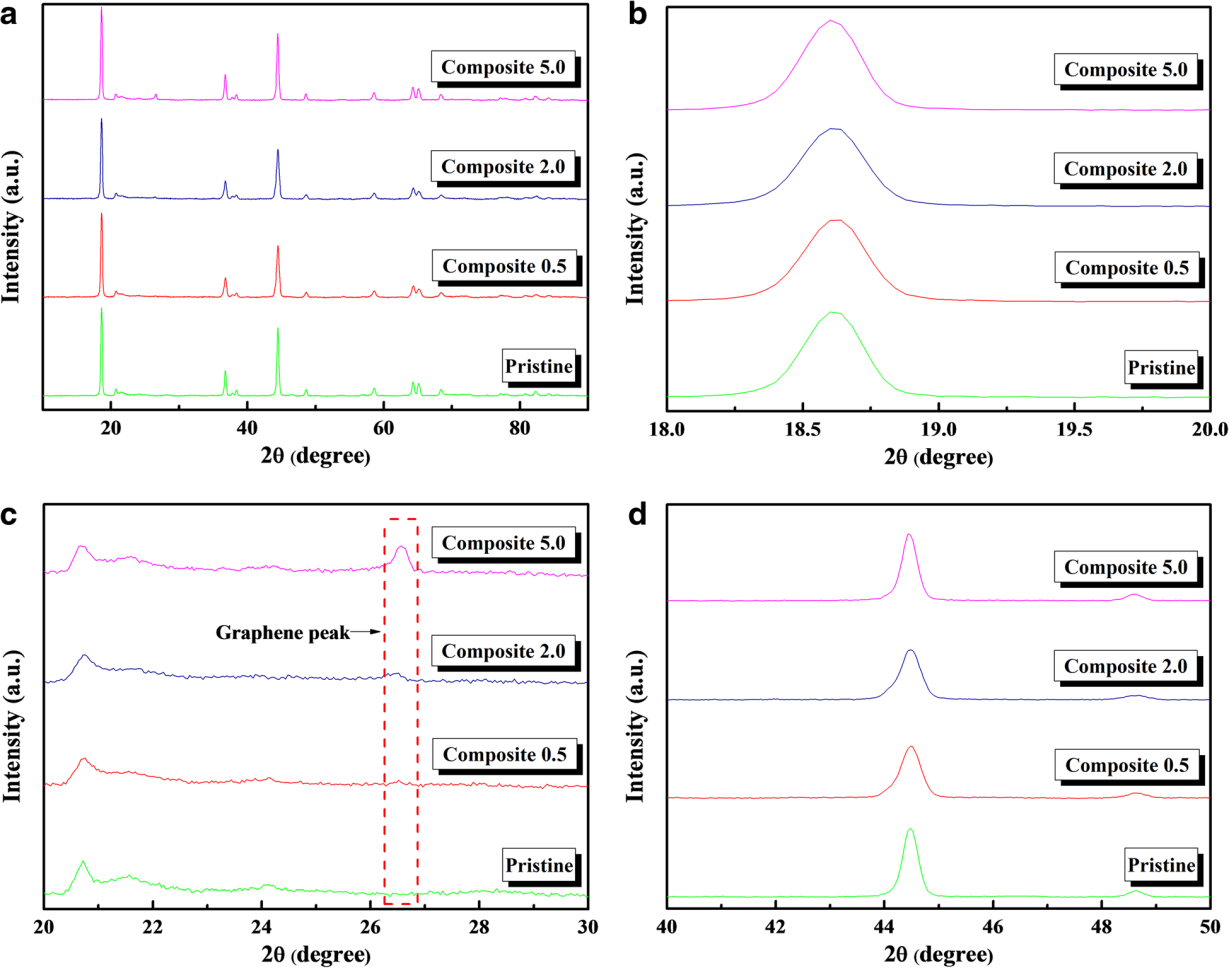
XRD patterns of the pristine and composite samples. **a** Full range (10°–90°), **b** 18°–20°, **c** 20°–30°, and **d** 40°–50°

As mentioned previously, we expected the Li[Ni_0.2_Li_0.2_Mn_0.6_]O_2_ particles in our composites to retain their structural integrity and phase; this assumption was deemed reasonable since the composites were assembled via electrostatic interaction, without the use of a high-temperature heat-treatment process that leads to oxygen loss and phase changes. To determine the validity of this assumption, the pristine sample and composites were evaluated via XRD and XPS measurements. Figure 4a compares the XRD patterns of the samples. The patterns (Fig. 4a) from the pristine sample and the composites were very similar and corresponded closely to that of the hexagonal α-NaFeO_2_ structure (space group R-3m); the peaks occurring at angles of 20°–25° are associated with superlattice ordering in the transition metal layers. The pattern was examined in further detail by enlarging the peaks occurring at angles of 18°–20°, 20°–30°, and 40°–50°. These peaks are associated with the (003), superlattice ordering and graphene, and (104) and (105) reflections, which are shown in Fig. 4b–d, respectively. Previous studies [28, 29] have shown that the diffraction pattern of carbon/Li-rich oxide composites (prepared via a heat-treatment process) differed somewhat from that of the pristine sample. There, the (003) peaks were shifted and peaks associated with the spinel-like phase occurred, owing to the effect of oxygen loss. However, in our work, the peaks corresponding to the pristine and composite samples were almost identical, indicating that the method of electrostatic interaction prevented the phase change of the vulnerable Li-rich oxide (Li[Ni_0.2_Li_0.2_Mn_0.6_]O_2_). As shown in Fig. 4c, the peaks corresponding to graphene are clearly detected in the patterns of the composites.

**Fig. 5:**
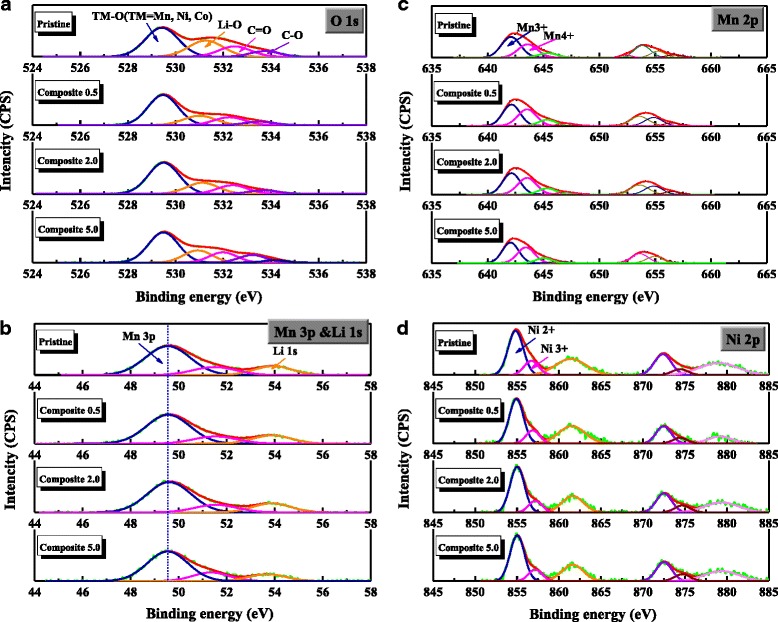
XPS spectra of the pristine and composite samples. **a** O 1*s*, **b** Li 1*s*, **c** Mn 2*p*, and **d** Ni 2*p*

Figure 5 shows the XPS spectra of the O 1*s*, Li 1*s*, Mn 2*p*, and Ni 2*p* orbitals of the pristine and composite samples. The curves were all calibrated based on the C 1*s* (C–C bond) peak (284.5 eV). The XPS spectrum of the composite prepared via a heat-treatment process differed significantly from that of the pristine sample [28]. Specifically, the intensity of TM-O (TM = transition metal such as Ni, Mn, Co) bond (~529.5 eV) was increased, and the intensity of Li 1*s* peak (~54.2 eV) was decreased. This indicates that compared to the pristine sample, the composites have higher and lower content of transition metal and Li, respectively, on their surface. Furthermore, this result is attributed to the phase transformation of the surface layer to the spinel-like (−LiMn_2_O_4_) phase; this phase has a higher transition metal (Mn) content and lower amounts of Li than the pristine Li-rich oxide (typically, the Li:Mn ratio is approximately 1.2:0.8). However, this phase transition did not occur in the composites prepared by our method of electrostatic interaction. The peaks corresponding to the TM–O (TM=Mn and Ni) bond (~529.5 eV) of the samples (Fig. 5a) exhibited similar intensities, although those of the C–O and C=O bonds increased with increasing C content of the composites. The peaks corresponding to the Li 1*s* (~54.2 eV) also exhibited (Fig. 5b) similar intensities in both the pristine and the composite samples. This indicates that the spinel-like phase did not form during the fabrication of the composites. Moreover, the peak corresponding to the Mn 3*p* and Mn 2*p* occurred in similar positions in both the pristine and composite samples, as shown in Fig. 5b, c. This indicates that the composites have similar Mn oxidation states as their pristine counterpart. Previous studies [28, 29] showed the Mn oxidation state of composites prepared via a heat-treatment process differed significantly from that of the pristine sample. The peaks related to Mn shifted, and the intensity of those was also changed due to composite-process. Therefore, the similarities observed in Fig. 5 are indicative of the phase integrity of our composites that are prepared by electrostatic interaction.

**Fig. 6:**
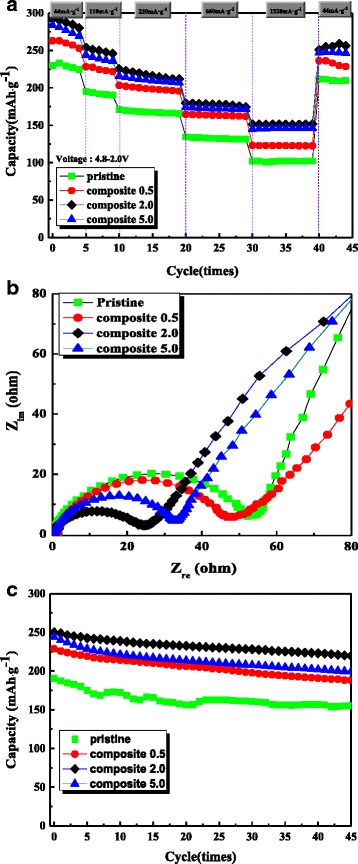
**a** Discharge capacities of the pristine sample and the graphene/Li[Ni_0.2_Li_0.2_Mn_0.6_]O_2_ nanoparticle composites at current densities of 44, 110, 220, 660, and 1320 mA g^−1^ in a voltage range of 4.8–2.0 V; **b** Nyquist plots of the pristine sample and the graphene/Li[Ni_0.2_Li_0.2_Mn_0.6_]O_2_ nanoparticle composites before electrochemical testing; **c** cyclic performance of the pristine sample and the graphene/Li[Ni_0.2_Li_0.2_Mn_0.6_]O_2_ nanoparticle composites at a current density of 110 mA g^−1^

The effect of graphene as a matrix material was determined by evaluating the electrochemical properties of the pristine samples and the composites. Figure 6a shows the discharge capacities of the samples measured at current densities of 44, 110, 220, 660, and 1320 mA g^−1^, in a voltage range of 4.8–2.0 V. As the figure shows, the discharge capacities of the composites are all somewhat higher than that of the pristine sample. Composites 2.0 and 5.0 have, in particular, a higher discharge capacity and superior rate capability compared with those of composite 0.5. Table 1 summarizes the discharge capacities and capacity retentions of the samples measured at various current densities (3rd, 6th, 11th, 21st, and 31st cycles of Fig. 6a). The capacity retention of the pristine sample at 1320 mA g^−1^ was only ~44 % of that measured at a current density of 44 mA g^−1^. In contrast, composites 2.0 and 5.0 exhibited superior capacity retention of ~53 %. These improved electrochemical properties stem from the effect of graphene acting as a matrix for the Li[Ni_0.2_Li_0.2_Mn_0.6_]O_2_ nanoparticles; i.e., the high electronic conductivity of the graphene matrix compensates for the low conductivity of the Li[Ni_0.2_Li_0.2_Mn_0.6_]O_2_ nanoparticles, thereby leading to the enhanced electrochemical performance of the composite.

**Table 1:** Discharge capacity and capacity retention of the pristine and composite samples at various current densities

Current density	44 mA g^−1^ (mAh g^−1^)	110 mA g^−1^ (mAh g^−1^)	220 mA g^−1^ (mAh g^−1^)	660 mA g^−1^ (mAh g^−1^)	1320 mA g^−1^ (mAh g^−1^)	Retention rate (%)
Pristine	229	194	170	134	101	44.1
Composite 0.5	260	228	203	164	123	47.3
Composite 2.0	287	253	225	179	151	52.6
Composite 5.0	276	243	215	174	145	52.5

The percentages refer to the retention of the capacity at the 31st cycle compared with that at the 3rd cycle in Fig. 6a

Figure 6b shows the results of electrochemical impedance spectroscopy measurements performed prior to the electrochemical tests. The Nyquist plots composed of a broad semicircle, which may be overlapped two semicircles. Generally, a semicircle located in high-frequency range represents the impedance due to a solid electrolyte interface, and a semicircle in relatively low-frequency range represents the charge-transfer resistance at the electrode/electrolyte interface [9, 30]. The size of the semicircle is dependent upon the impedance value of the cell. As shown in Fig. 6b, the semicircles associated with the composites had smaller diameters than the semicircle corresponding to the pristine sample. This indicates that graphene-containing composites are effective in reducing the impedance value of the Li[Ni_0.2_Li_0.2_Mn_0.6_]O_2_ cathode. Furthermore, the enhanced rate capability (Fig. 6a) of the composites results from this reduced impedance. The impedance value of the composite 5.0 was somewhat higher than composite 2.0, which may due to large amount of graphene. Too much graphene can block Li^+^ transport between liquid electrolyte and cathode surface since Li^+^ cannot penetrate through the graphene layer.

**Fig. 7:**
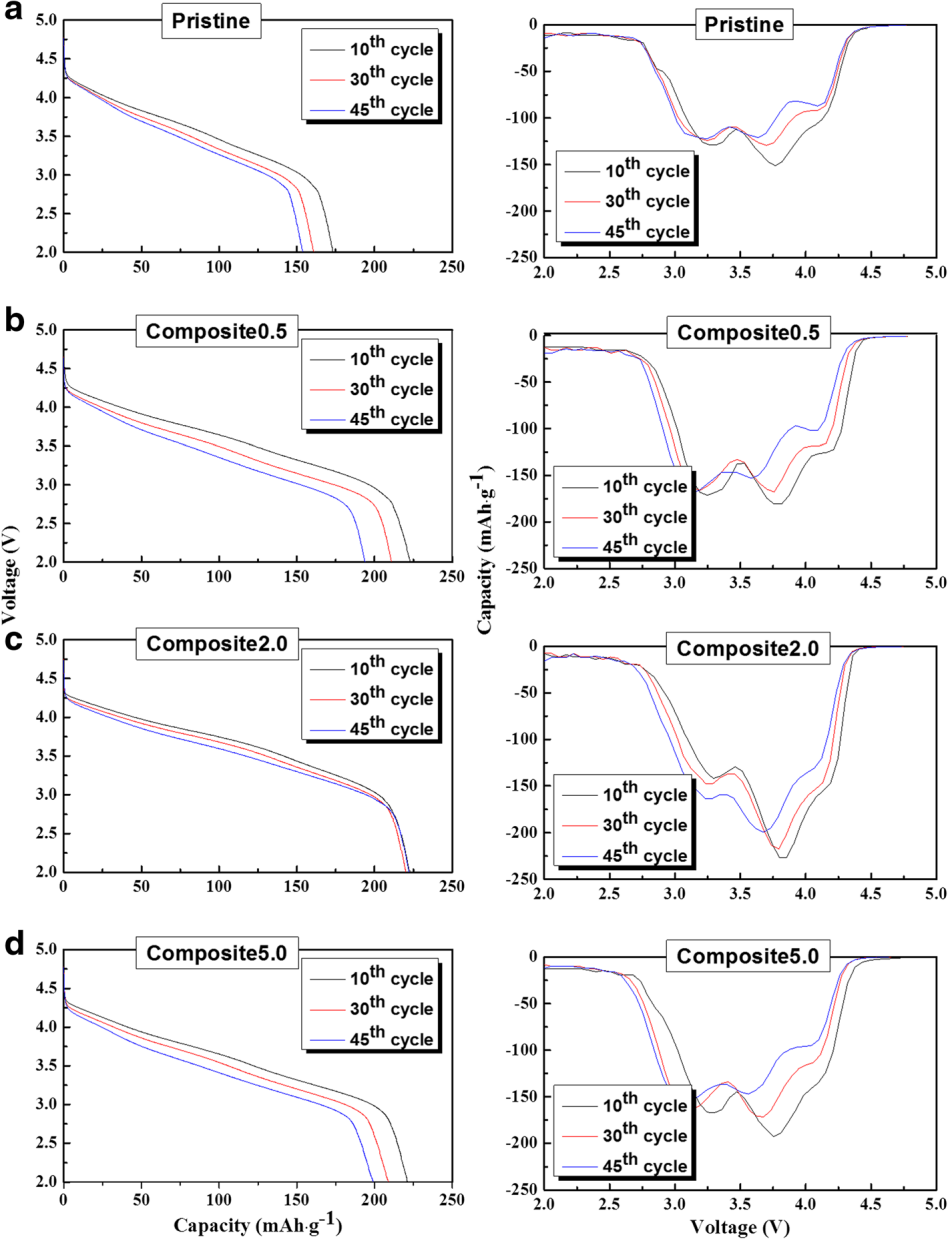
The 10th, 30th, and 45th discharge profiles and dQ/dv plots of the samples in Fig. 6c. **a** Pristine, **b** composite 0.5, **c** composite 2.0, and **d** composite 5.0

Figure 6c shows the cyclic performance of the samples measured at a current density of 110 mA g^−1^ and a voltage range of 4.8–2.0 V. The discharge capacity of the composites was somewhat higher than that of the pristine sample. However, the cyclic performance of the samples differed only slightly. The corresponding discharge profiles after various cycles (left in Fig. 7) reveal that the discharge capacities of the samples decrease gradually during cycling. More importantly, a double-plateau region did not form during cycling. These plateaus are indicative of the phase transformation from a layered structure of Li-rich oxides to a spinel-like structure, during cycling of the carbon/Li-rich oxide composite fabricated via a heat-treatment process [28, 29]. This transformation is attributed to the deterioration of phase integrity and the oxygen loss stemming from the reaction with C (graphene) during the fabrication process. However, the absence of these plateaus indicates that our composite, prepared by electrostatic interaction, maintained a stable phase during cycling; i.e., the compositing process did not reduce the phase integrity of the Li-rich oxide. This can be also confirmed by the dQ/dV plots in Fig. 7 (right side). The peaks in the samples shifted to the low potential during cycling. However, the sharp peak below 3.0 V, indicating the phase transformation to spinel [28, 29], was not growing during cycling. Therefore, the novel fabrication method described in this work constitutes an effective approach for maintaining phase integrity and enhancing the electrochemical performance of Li-rich oxides that consist of a vulnerable phase.

## Conclusions

Li[Ni_0.2_Li_0.2_Mn_0.6_]O_2_ nanoparticles were successfully composited on the surface of graphene using electrostatic interaction without a high-temperature heat-treatment process. Carbon/Li-rich oxide composites (prepared via a heat-treatment process) are, in general, susceptible to phase changes and deterioration of the phase integrity; this deterioration results from the reaction of carbon with oxygen and consequent loss of oxygen from the structure of the vulnerable Li-rich oxide phase. However, we successfully fabricated graphene/Li[Ni_0.2_Li_0.2_Mn_0.6_]O_2_ composites using electrostatic interaction without deterioration of the phase integrity of the oxides, as confirmed by XRD and XPS analysis. The optimized graphene/Li[Ni_0.2_Li_0.2_Mn_0.6_]O_2_ composites (composite 2.0) exhibited higher discharge capacity and improved rate capability compared with those of pristine Li[Ni_0.2_Li_0.2_Mn_0.6_]O_2_. This improvement is attributed to the high conductivity of graphene, which compensates for the low conductivity of the pristine Li[Ni_0.2_Li_0.2_Mn_0.6_]O_2_.
